# Effect of synaptic plasticity on functional connectivity and global activity of a neocortical network model

**DOI:** 10.1186/1471-2202-16-S1-P210

**Published:** 2015-12-18

**Authors:** Renan O Shimoura, Rodrigo FO Pena, Antonio C Roque

**Affiliations:** 1 Departamento de Física, FFCLRP, Universidade de São Paulo, Ribeirão Preto, SP, Brazil

## 

Neocortex plays key role in diverse brain functions. Understanding this role involves the study of collective neural activity patterns under different situations, and how these patterns relate to the structural and functional organization of neocortex. Here we study the effect of synaptic plasticity on neural spiking activity patterns in a neocortical network model. We measure changes in neural spiking patterns due to changes in the strengths of the synapses connecting neurons and relate them to changes in the functional connectivity of the network as disclosed by graph-theoretic measures.

Our neocortical network model was composed of excitatory and inhibitory neurons in the proportion of four excitatory cells for each inhibitory cell. Neurons were described by the Izhikevich model [1]. The parameters of the model were adjusted so that excitatory neurons were of the regular spiking (RS) type and inhibitory neurons were all of either the fast spiking (FS) or the low-threshold spiking (LTS) type. Synapses were modeled as event-based, and two types of synaptic dynamics were considered: one without synaptic plasticity in which the synaptic weight received a fixed increment after the pre-synaptic event and decayed exponentially after that, and one with synaptic plasticity in which the synapse obeyed an asymmetric spike-timing dependent plasticity (STDP) rule described by [2]. Neurons were organized into four layers (2/3, 4, 5 and 6) with layer- and cell-specific statistical connectivity rules based on [3]. The total number of neurons in the model was about 4,000. Two experiments were done: one with all synapses described by the model without synaptic plasticity, and the other with synapses between excitatory neurons described by the STDP rule while the remaining synapses were described by the model without synaptic plasticity. In both cases, the model was stimulated by a current injection of random amplitude applied to neurons of layer 4 (L4), which is the main input layer of the cortex. The spiking activity of the network was evaluated by measures extracted from the raster plot of the spikes produced by the neurons, e.g. layer-specific and network mean and time-dependent firing rates. The structural and functional connectivities of the network were represented by the respective structural and functional adjacency matrices. The functional adjacency matrix was constructed by taking in consideration neuron pairs with strength of their synaptic coupling above a specific threshold. The topology of the adjacency matrices was characterized by graph-theoretic measures, e.g. clustering coefficient.

We determined a set of parameters for which the spiking activity generated in L4 by the external input propagated to the entire network. This network-wide activity was oscillatory, and we found that its mean frequency was higher for the network version with synaptic plasticity than for the version without synaptic plasticity. We also found that the formation of clusters of synchronous neural activity was facilitated in the case with LTS cells as inhibitory neurons. Our results suggest that synaptic plasticity may induce changes in the functional connectivity of the neocortical network with impact on its global activity.
